# The use of co-production, co-design and co-creation to mobilise knowledge in the management of health conditions: a systematic review

**DOI:** 10.1186/s12913-022-08079-y

**Published:** 2022-07-07

**Authors:** Cheryl Grindell, Elizabeth Coates, Liz Croot, Alicia O’Cathain

**Affiliations:** 1 Health and Care Research Unit, School of Health and Related Research (ScHARR), The University of Sheffield, Sheffield, UK; 2 Clinical Trials Research Unit, School of Health and Related Research (ScHARR), The University of Sheffield, Sheffield, UK

**Keywords:** Co-production, Co-design, Co-creation, ‘Co’approaches, Knowledge mobilisation, Health

## Abstract

**Background:**

Knowledge mobilisation is a term used in healthcare research to describe the process of generating, sharing and using evidence. ‘Co’approaches, such as co-production, co-design and co-creation, have been proposed as a way of overcoming the knowledge to practice gap. There is a need to understand why researchers choose to adopt these approaches, how they achieve knowledge mobilisation in the management of health conditions, and the extent to which knowledge mobilisation is accomplished.

**Methods:**

Studies that explicitly used the terms co-production, co-design or co-creation to mobilise knowledge in the management of health conditions were included. Web of Science, EMBASE via OvidSP, MEDLINE via OvidSP and CINHAL via EBSCO databases were searched up to April 2021. Quality assessment was carried out using the Joanna Briggs Institute qualitative quality assessment checklist. Pluye and Hong’s seven steps for mixed studies reviews were followed. Data were synthesised using thematic synthesis.

**Results:**

Twenty four international studies were included. These were qualitative studies, case studies and study protocols. Key aspects of ‘co’approaches were bringing people together as active and equal partners, valuing all types of knowledge, using creative approaches to understand and solve problems, and using iterative prototyping techniques. Authors articulated mechanisms of action that included developing a shared understanding, identifying and meeting needs, giving everyone a voice and sense of ownership, and creating trust and confidence. They believed these mechanisms could produce interventions that were relevant and acceptable to stakeholders, more useable and more likely to be implemented in healthcare. Varied activities were used to promote these mechanisms such as interviews and creative workshops. There appeared to be a lack of robust evaluation of the interventions produced so little evidence in this review that ‘co’approaches improved the management of health conditions.

**Conclusion:**

Those using ‘co’approaches believed that they could achieve knowledge mobilisation through a number of mechanisms, but there was no evidence that these led to improved health. The framework of key aspects and mechanisms of ‘co’approaches developed here may help researchers to meet the principles of these approaches. There is a need for robust evaluation to identify whether ‘co’approaches produce improved health outcomes.

**Trial Registration:**

PROSPERO CRD42020187463.

**Supplementary Information:**

The online version contains supplementary material available at 10.1186/s12913-022-08079-y.

## Background

The term ‘knowledge mobilisation’ is used in the healthcare literature to describe the active, iterative and collaborative process of creating, sharing and using research evidence [1, 2]. Ideally all forms of knowledge, such as experience, values and beliefs are considered in this process—not just scientific factual knowledge [3, 4]. This is in contrast to the term ‘evidence’ where patients’ voices are considered bottom of the evidence hierarchy [4]. Research and healthcare practice inhabit very different worlds, with contrasting goals and using different languages [4]. A shift from hierarchical models of evidence, that favour scientific/medical knowledge, to other forms where patient voice is more at the forefront has been recommended [4]. This has led to a change from linear, rational approaches to knowledge mobilisation to more disordered, relational, context driven ones [4, 5]. Knowledge mobilisation as a concept remains confusing and is often considered an umbrella term for other forms of knowledge sharing and use such as knowledge translation, exchange and dissemination [3, 5, 6]. These terms are frequently used interchangeably within the literature.

Involving patients and clinicians in the generation of new knowledge is considered important to ensure research findings are impactful and to reduce research waste [7, 8]. The need to make public services evidence-based remains of high importance [5] in order to improve the management of health conditions such as cardiovascular disease, osteoarthritis and cancer. Many of these health conditions require long term management that place high burden on healthcare services [9]. Sharing and generating knowledge between patients and clinicians can help improve understanding of living with and treating these conditions. This can positively impact disease progression, burden of care and health outcomes [9]. However involving patients and clinicians in research or service improvement is challenging and sometimes tokenistic [7]. Social hierarchies exist which means not all knowledge is valued and considered equally [10]. Co-creative approaches to knowledge production have been advocated to bridge the knowledge-to-practice gap [5, 8]. There are many different collaborative and participatory methods in the health research and service improvement literature [7], with a multitude of approaches being used. Co-production, co-design and co-creation are common terms; these terms have been summarised as ‘co’approaches [11]. The fundamentals of ‘co’approaches have been described in the literature, for example the UK’s National Institute for Health Research (NIHR) principles for co-production [12]. Despite this, there is little consensus about the type of approaches the three terms describe [11, 13]. Common uses of these terms are: 1) co-production of a research project where researchers, practitioners and the public work together throughout the course of the project [12]; 2) co-creation of new knowledge by academics working alongside other stakeholders [8] and; 3) co-design when developing complex interventions [14]. In practice, the three terms are often used interchangeably and adopted and described inadequately and ambiguously [11, 15]. Many ‘co’approaches do not address the egalitarian and utilitarian values of what is considered ‘genuine’ co-production leading to a crowded landscape of terms and approaches beginning with the word ‘co’ that Williams et al. (2019) have described as ‘cobiquities’ [13].

There is currently a lot of interest in knowledge mobilisation and ‘co’approaches in health, with multiple publications about their use. Several reviews have explored the use of specific co-production, co-design or co-creation processes. A recent review undertook content analysis of the co-creation of knowledge for health interventions aiming to reduce the term’s ambiguity and provide a clear definition [15]. The authors developed a new evidence-based definition of knowledge co-creation but included a number of other ‘co’ terms within this, still leaving the reader to address a confusing landscape of ‘cobiquities’. A rapid review of research co-design in health settings had a specific focus on the planning stages of a research project only [16]. Another review sought to understand the outcomes associated with developing and implementing co-produced interventions in acute healthcare settings [17]. The latter reported findings related to understanding the processes of co-designing a service rather than evaluating outcomes themselves. They found different forms of co-production were reported, often uncritically, with a lack of consistent use of terminology to support this diverse range of participatory approaches [16, 17].

To the authors’ knowledge there has yet to be a systematic review that has specifically explored the use of ‘co’approaches in knowledge mobilisation in the management of health conditions. This systematic review aimed to explore why researchers use ‘co’approaches, how researchers think ‘co’approaches can achieve health improvement, the activities they use, and whether they achieve knowledge mobilisation in the management of health conditions (actual or perceived).

## Methods

This is a mixed studies systematic review, that is, a comprehensive review and synthesis of a wide range of literature of diverse designs [18]. Mixed studies reviews are useful for understanding complex phenomena such as ‘co’approaches for knowledge mobilisation. Seven standard systematic review steps for mixed studies reviews have been followed [18]: 1. Writing a review question. 2. Defining eligibility criteria. 3. Applying an extensive search strategy in multiple information sources. 4. Identifying potentially relevant studies (by two independent researchers screening titles and abstracts). 5. Selecting relevant studies (based on full text). 6. Appraising the quality of included studies using an appropriate tool. 7. Synthesising included studies.

Conduct and reporting of the review followed the Preferred Reporting Items for Systematic reviews and Meta Analysis checklist and flow chart to ensure transparency and complete reporting of the findings [19]. The review was registered with PROSPERO (registration number CRD42020187463 September 2020).

### Review questions

1. What is the rationale for using ‘co’approaches to mobilise knowledge in the management of health conditions?
2. What mechanisms of ‘co’approaches achieve knowledge mobilisation (actual or perceived) in the management of health conditions?
3. What type of activities are used within ‘co’approaches to mobilise knowledge in the management of health conditions?
4. To what extent do ‘co’approaches achieve knowledge mobilisation (actual or perceived) to help manage health conditions?

### Defining eligibility criteria

Specific inclusion and exclusion criteria were defined using the PICOS framework, Population, Intervention, Context, Outcome and Study type [20]. See Table 1. One of three common terms, that is co-production, co-design and co-creation, had to be explicitly used in a paper for inclusion in this review.

**Table 1:** Inclusion and exclusion criteria

Inclusion criteria	Exclusion criteria
**Population** Children, adults, patients, carers, healthcare staff and researchers **Intervention** Explicit use of co-design, co-production or co-creation to mobilise knowledge, where knowledge mobilisation includes the generation, sharing, transformation and use of knowledge/evidence in practice **Context** All studies investigating a health condition including acute care, sub-acute care, community health and non-health settings delivering health-related activities **Study type** Primary research, either, quantitative, qualitative or mixed methods (including study protocols), case studies, commentary and discussion and opinion papers and grey literature Studies published in English	**Population** Non-human participants **Intervention** Studies where the knowledge mobilisation strategy is not explicitly termed co-design, co-production or co-creation Patient and public involvement in research, and collaboration and participatory approaches unless specifically described as co-production/design/creation **Context** Studies not focused on management of a specific health condition **Study type** Studies not published in English

### Applying an extensive search strategy in multiple information sources

#### Systematic search of academic literature

Searches were conducted of four electronic databases: Web of Science (all databases) 1970—April 2021, EMBASE via OvidSP 1988 – April 2021, MEDLINE via OvidSP 1946 – April 2021, CINHAL via EBSCO 1981—April 2021. Initial full database searches were carried out up to 26^th^ May 2020. Search alerts were used from this point on for all four databases up until the end of April 2021. The University of York’s Centre for Reviews and Dissemination database, the Cochrane Library (CENTRAL) and Trip medical database were also searched. Bibliographic searches of selected articles reference lists were browsed for any additional relevant studies [21].

### Structured search of the grey literature

Grey literature (unpublished) searches were also conducted to identify any literature from non-traditional sources and to minimise publication bias [21]. Grey literature sources such as Open Grey and Google were conducted as well as websites of professional networks in the field, for example the Canadian Integrated Knowledge Translation (IKT) Network. It is acknowledged that a google search may produce many pages of potentially relevant literature. In this case the first eight pages of the google search were screened. At which point the number of relevant literature significantly diminished. Publications situated on the university profile pages of academic experts in co-production and or knowledge mobilisation were also searched. These were identified through a UK Knowledge Mobilisation Alliance and through recommendations of academic peers. Citation searching from the reference lists of included studies was also carried out.

### Search terms

A comprehensive search strategy was developed in conjunction with an information specialist and was performed by the primary reviewer (CG). A wide variety of key search terms, based on terms in the review question, were used. They included free text and subject headings (such as MeSH) where appropriate. Truncationfor certain key words was used for completeness. Boolean logic operators AND / OR were then utilised to combine terms [21]. For example:

1. Co-production OR co-prod* OR coproduction OR coproduc* OR co production OR co produc*OR codesign OR co-design OR co design OR co-creat* OR cocreat* OR co creat*
2. AND
3. Knowledge mobil* OR Knowledge transl*OR knowledge utili*OR knowledge exchange OR knowledge uptake OR Knowledge to action OR Knowledge to practice OR Evidence based practice.

Search terms were purposely limited to try and provide some focus on what is a very crowded and complex landscape. Multiple terms are often used in the literature for co-productive activities which can be confusing. This systematic review purposely sought to provide some clarity on the use of the three common ‘co’ terms, co-production, co-design and co-creation rather than, for example patient and public involvement and engagement. The same can be said for knowledge mobilisation. Therefore this study limited the use of knowledge mobilisation terms to those frequently seen in the healthcare literature and which encompassed a more interactional, two way flow of knowledge. Implementation was specifically not used, even though it could be argued it is the final stage of knowledge mobilisation, so not to cause confusion between these two different but similar terms and their meanings.

See supplementary material 1 (word document) for detailed search terms used.

### Identifying relevant studies

All database search results were imported and organised in Endnote X8 and exported to an Excel spreadsheet. Duplicate references were removed. This selection process allowed for transparency and reproducibility [21]. Documents were screened by title and then by abstract using the pre-determined eligibility criteria. Any articles that appeared to fulfil the inclusion criteria were obtained in full [20, 22, 23]. One reviewer (CG) screened all citations by title and abstract and a second reviewer (EC) independently screened 50. A high level of agreement was achieved between CG and EC on initial screening (90%). The remaining 10% were uncertainties mainly on CG’s part, who was an early career researcher. These uncertainties were resolved through discussion with EC, a more experienced researcher. It was therefore agreed, due to the high level of initial agreement and lessons learnt through the discussions, that the process was robust enough for CG to review the remaining titles and abstracts.CG then assessed the full text of all potentially eligible studies and EC reviewed 20% of the full text articles. EC provided a second opinion for papers CG was unclear about. CG and EC discussed any uncertainties and disagreements and reached a consensus on which studies to include.

### Data extraction and management

A standardised data extraction form was developed and tested on a small number of selected studies and then refined [20, 23]. The type of data extracted included: study characteristics such as type of study, setting, participant characteristics, rationale given by researcher for using a ‘co’approach, proposed mechanisms of ‘co’approach, type of activities used and outcomes of ‘co’approach (measured or perceived impact on knowledge mobilisation). The first reviewer (CG) extracted the data from all the included studies and a second reviewer (EC) double extracted 20% of papers to ensure consistency.

### Appraising the quality of included studies

There was a mixture of study types in this review including qualitative studies, co-design case studies and study protocols. Five of the 24 papers were mixed methods with qualitative research dominance, that is, they collected survey data alongside the main qualitative findings. The Joanna Briggs Institute (JBI) quality assessment checklists were chosen as they cover a variety of study designs [23]. Due to the nature of the included studies, the JBI qualitative quality assessment check list was used for all studies as a ‘best fit’. This was because there are no specific checklistsfor study protocols and case studies. Studies were not excluded based on quality as long as they addressed the focus of the review. This was to ensure no rich and meaningful insights from the data were lost [24]. CG appraised all selected studies and EC double appraised 20% of the selected studies. Any disagreements were resolved through discussion.

### S*ynthesising included studies*

A thematic synthesis approach was used based on the principles of Thomas and Harden (2008) [25]. This has three stages: line by line coding of text, development of descriptive themes, and generation of analytical themes [25]. Analytical themes were not relevant for all the research questions so descriptive themes are presented. NVivo QSR (2020) was used to store and organise the extracted data. There was a small amount of quantitative data extracted in this review in the form of descriptive statistics. A convergent integrated approach was used [23, 26]. The quantitative data was ‘qualitized’ and turned into textual descriptions and then combined with the qualitative data [23, 26]. This allowed for a narrative interpretation of the quantitative results [23].

## Results

### Characteristics of studies

**Fig. 1:**
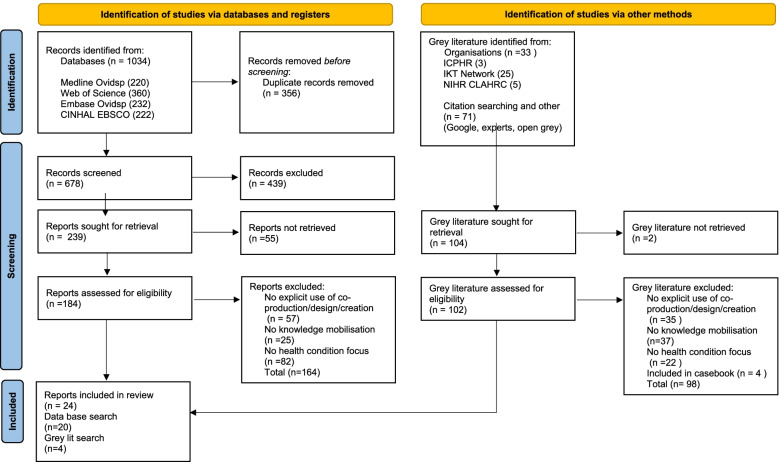
PRISMA 2020 flow diagram [19]

The searches identified 1171 studies. After deduplication 782 were screened by title and abstract. This was a challenging task due to the broad and varied use of the terms co-production, co-design, co-creation and knowledge mobilisation in the literature. The remaining 286 articles were reviewed in full text to assess their eligibility, resulting in 24 included in the review. See Fig. 1.

Study characteristics are shown in Table 2. The included studies were conducted internationally: in the UK (*n *= 9) [27–35], Australia (*n* = 7) [36–42], Canada (*n* = 5) [43–47], Sweden (*n* = 2) [48, 49] and Italy/UK (*n* = 1) [50]. The majority of the studies were qualitative case studies [27–29, 32, 33, 35, 36, 38–41, 50]. Five of these studies collected and presented survey data alongside the narrative data [30, 42, 43, 48, 49]. Three papers were qualitative study protocols [31, 37, 47]. One was a patient-led (co-designed) qualitative study [46], and there were three case study collections [34, 44, 45]. Numbers of participants varied across studies from 7- 156. All three terms co-production [28, 29, 32–35, 50], co-design [28, 30, 31, 34, 37–41, 43, 44, 48, 49] and co-creation [36, 45, 47], were used to define their knowledge mobilisation approach.

**Table 2:** Study characteristics

Author, year, country	Health condition	Aim of study	Methodology	Participant numbers	Co-approach	Definition of co-approach used by authors
Cowdell et al. (2020) UK [27]	Eczema	To devise strategies to amend lay and practitioner eczema mindlines to improve consultation experiences and self- management practices in primary care. To identify 1. What knowledge needs to be mobilised. 2. Who needs this knowledge. 3. How should this knowledge be shared	Qualitative case study	Total n = 22 Lay people n = 10 Health practitioners n = 12	Co-Creation	Using 8 principles of Co-Create co-production matrix: Holistic, resourced, transparent, inclusive, iterative, positive, equal, Sustainable
Dal Mas et al. (2020) Italy/UK [50]	Breast cancer	How can knowledge translation be triggered by design to support and enhance the physical and psychological recovery of patients after breast cancer surgery	Qualitative case study	Total n = 28 Researchers n = 4 Psychiatrists/physiotherapists n = 9 Nurses n = 3 Breast surgeon n = 1 Sport and fitness professional n = 1 Patients n = 2 National association of breast surgery operated women n = 2 Librarians n = 3 Admin staff n = 3	Co-production	Patient engagement Active and effective participation of patients in their healthcare
Dent et al. (2016) Australia [36]	Long term musculoskeletal problems	Describe lessons learned from implementation of a population health intervention study in a rural setting using a Co-KT framework as a guideline for intervention	Qualitative case study	Not reported	Co-creation (cocreating a knowledge translation framework – Co-KT)	‘Co-creating of KT’ (Co-KT) framework, which combines academic evidence-based knowledge with the context-specific knowledge from stakeholders
Fonseka et al. (2019) Canada [43]	Mental Health	A knowledge translation project to adapt the CANMAT clinician guidelines into an accessible, plain language version	Qualitative case study	Total n = 7 Workshop People with lived experience of mental health problems n = 7	Co-design	Incorporating expertise of individuals with lived experience
Grindell et al. (2020) UK [28]	Malignant pleural effusion	To describe the co-design methods used to mobilse knowledge and co-create a decision support tool for people with malignant pleural effusion	Qualitative case study	Total n = 41 Workshop 1 Site 1 Total n = 9, Consultant physician n = 3 Patients n = 5 Carers n = 2 Nurse specialist n = 1 Site 2 Total n = 11, Consultant physician n = 1 Physician Registrar n = 1 Patients n = 5 Carers n = 3 Nurse specialist n = 1 Research nurse n = 1 Site 3 Total n = 11, Physician registrar n = 1 Patients n = 5 Carers n = 5 Senior research nurse n = 1 Student nurse n = 1 Workshop 2 Total n = 10 Consultant physicians n = 2 Physician registrar n = 3 Nurses n = 3 Patients n = 2	Creative co-production/design	A four phased, human-centred process of divergent and convergent thinking. Recognising all forms of knowledge. Considering all ideas before the best, most practical solutions are tested through an iterative prototyping process ready for implementation
Heaton (2016) UK [29]	Acute stroke management	What does the theory of co- production add to our understanding of the processes of knowledge creation and translation in PenCLAHRC	Qualitative case study	Total n = 9 NHS trust staff and local stroke network n = 5 Researchers n = 4	Co-production	Co-production of knowledge and closer collaboration
IKT casebook vol 1 (2019) [44] Case studies: Townley et al., Sibbald et al., Gainforth et al., Kastner et al Editors McCutcheon et al Canada	4 case studies includes chronic pain assessment, spinal cord injury and multi chronic disease	Using a integrated knowledge translation approach to co-create a pain assessment toolkit, and physical activity interventions and to co-design a multi disease management tool	Case studies	Not disclosed	Co-production, co-creation and co-design	Not explicitly defined beyond an integrated knowledge translation approach
IKT casebook vol 3 (2020) [45] Case study: Ramage et al Editors Boland et al	Stroke	The co-design and piloting of an evidence-based intervention aimed at increasing physical activity to reduce secondary stroke risk	Case study	Total n = 45 Knowledge user partners Total n = 13 Person with lived experience of stroke n = 1 Physiotherapists n = 2 Exercise scientist n = 1 Researchers n = 5 PhD supervisors n = 4 (with research expertise in physiotherapy [n = 3] and nutrition and dietetics [n = 1]) Knowledge-user informants Total n = 32 Health-care workers (n = 16) such as doctors, nurses, physiotherapists, managers Stroke survivors (n = 10) Carers (n = 5) Behaviour change researcher (n = 1)	Co-design	Not explicitly described but involving knowledge user partners and knowledge user informants at each stage of project
Knowles et al. (2018) UK [30]	People with multi-morbidity	To explore whether co- production methodologies could enhance intervention development and provide a mechanism to translate available evidence into patient- centred intervention proposals for multimorbidity and safety	Qualitative (codesign and survey)	Total n = 34 Workshop 1 Total n = 11 People or carers with multi morbidities n = 11 Workshop 2 Total n = 5 GP n = 1 Pharmacists n = 3 Pharmacy dispenser n = 1 Workshop 3 Total n = 11 Public contributors n = 9 Pharmacist n = 1 Pharmacy dispenser n = 1 Survey n = 7 Patients n = 4 Health care professionals n = 3	Co-design (participatory design)	Methodologies which explicitly involve patients in design and development
Law (2020) UK [31]	Long term conditions	To identify and produce a taxonomy of physical activity interventions that aim to reduce functional decline in people with long- term conditions managed in primary care (Stage 4 Intervention co-design, actionable recommendations and knowledge mobilisation)	Study protocol – realist synthesis with embedded co-production and co-design	Participant numbers not described	Co-design/production	Draw on the lived experiences of service users and professionals providing services to them. Ensuring all views from stakeholders are included and embedded within the process
Lewando Hundt et al. (2019) UK [32]	End of life care	Evaluation of research based theatre performance post discussions to capture the nature and dynamics of the co-production of knowledge	Qualitative case studies	Total n = 25–75 On average 50% 0f audience (n = 50–150) attended post show activities included service users, carers, students, researchers, and health, and social care service providers and the wider public	Co-production (of knowledge)	This term recognizes that the process involves multiple types of knowledge and experience from a plurality of stakeholders and actors
Livings et al. (2020) Australia [37]	Osteo-Arthritis	To establish whether a co-designed, community- based, physiotherapy- led multidisciplinary model of care for managing knee OA can be developed and implemented in the community physiotherapy setting	Study protocol a quasi- experimental, pre– post design with an embedded qualitative component- phase 2 = co-design	Aim to recruit 52	Co-design	Consultation with researchers, patients, clinical staff, members of the public and other stakeholders
Miller et al. (2016) Canada [46]	Osteo -Arthritis(OA)	What does quality care mean to patients with OA and what is most helpful in managing their arthritis	Qualitative	People with OA n = 25	Co-design	Co-design of research project- participants setting research questions, collecting data etc
Milton et al. (2021) Australia [38]	Mental health /eating disorders	To collaboratively customise and configure the InnoWell Platform to enhance access to and service quality of Butterfly’s National Helpline	Qualitative case study	Total n = 45 People with experience of eating disorders Workshop 1 n = 9 Workshop 2 n = 7 Workshop 3 n = 11 Workshop 4 n = 5 Workshop 5 n = 5 Workshop 6 n = 8	Co-design/participatory design	The active participation of all stakeholders to ensure that the end product meets the needs of its intended user base, improves usability, and increases engagement of all individuals
Ospina- Pinillos et al. (2018) Australia [41]	Mental health	To codesign and build a Mental Health eClinic (MHeC) to improve timely access to, and better quality, mental health care for young people across Australia	Qualitative case study	Total n = 44 Stage 1 n 28 Young people (YP) with mental health problems n = 18 Health care professionals (HCP) n = 10 Stage 2 n = 9 YP n = 6 HCP n = 4 Stage 3 n = 6 YP n = 4 HCP n = 2	Codesign (participatory design)	Involves iterative design cycles in which end users and researchers contribute to knowledge production and the development of the end product
Ospina- Pinillos et al. (2019) Australia [39]	Mental health	To co-design and culturally adapt the MHeC for Spanish-speaking young people based in Australia;	Qualitative case study	Total n = 32 Workshops n = 17 YP n = 10 HCP n = 7 User testing n = 15 YP n = 7 HCP n = 5 Supportive others n = 3	Codesign (participatory design)	involve stakeholders and end users in the design and development to increase user engagement and system usability
Ospina- Pinillos et al. (2020) Australia [40]	Mental health	To culturally adapt the MHeC for young people in Colombia	Qualitative case study	Total n = 28 Workshop n = 18 YP n = 7 HCP n = 11 User testing YP n = 5 HCP n = 3 Supportive others n = 2	Codesign (participatory design)	The process involves engaging end users and other stakeholders at all stages (from conception to completion) of the design, development, and testing of these technologies
Reeve (2016) UK [33]	Mental health and wellbeing	The aim was to translate a model of care into practice-based evidence describing delivery and impact. (started as a formative evaluation but finished as a co-production model)	Qualitative case study	Numbers not specified Initial evaluation: GP practices = 7 Redesign of intervention: GP practice n = 1	Co-production	To generate practice based knowledge to contextualise a complex intervention ready for implementation
Revenas (2018) Sweden [48]	Parkinsons Disease	The aim of this study was to describe the co-design an eHealth service for co-care (knowledge exchange) for Parkinson disease	Qualitative	Total n = 25 4 workshops: People with Parkinsons Disease n = 7 HCP n = 9 Facilitators n = 7	Co-design	Co-creation has been broadly defined as any act of collective creativity, while co-design signifies the span of a design process
Thompson (2020) Canada [47]	Functional constipation in children	To use patient engagement methods to establish a research collaboration with parents to co-create a digital knowledge translation tool for parents caring for a child with functional constipation	Qualitative study protocol	Specific numbers not disclosed	Co-creation	Not explicitly described but to be achieved through a parent collaborator group
Wannheden (2020) Sweden [49]	Parkinsons disease	This study explores People with Parkinson’s (PwP) and HCPs’ expectations and desired eHealth functionalities to achieve co-care (knowledge exchange to improve healthcare outcomes)	Qualitative (Co-design workshops and questionnaire)	Total n = 53 4 workshops n = 16 PwP n = 7 HCP’s n = 9 Prototype questionnaire n = 37 PwP n = 31 informal care givers n = 6	Co-design/participatory design	Participatory design shares similarities with action research and offers a method for combining health service and technology development in close collaboration with the intended users of the future service
Wolstenholme, Poll, Tod (2020) UK [35]	Hepatitis C	To devise interventions to improve access to the nurse-led hepatitis C clinic through sharing knowledge from those who both receive and deliver services	Qualitative case study	Total n = 22 Over 2 workshops: service users who were current or former patients of the hospital HCV clinic n = 12 Stakeholders representing seven different agencies n = 10	Co-production	Meaningful engagement of all stakeholders in the design of new services or knowledge. Ensuring the research is relevant to the end users and informed by them
Wolstenholme, Grindell, Tod, Bec (2018) UK [34]	Various health conditions including low back pain, chronic obstructive pulmonary disease, stroke	Highlights of how knowledge is translated, in its many forms, into action. With a particular focus on the contribution of creative practices and design to deliver successful change	Collection of case studies	Varies across projects From n = 10 – n = 75	Co-design	That allows the contribution of all the stakeholders of a project or service to share and synthesise new knowledge
Yeganeh et al. (2021) Australia [42]	Early menopause (EM)/ premature ovarian insufficiency (POI)	To describe and summarize the overall process of co-design and report on the development and evaluation of the digital resource as well as dissemination and implementation	Qualitative case study	Total 156 Interviews Women with EM n = 30 Surveys n = 126 Women with POI n = 110 HCP n = 16	Co-design	With all stakeholders including active patient inclusion, to ensure developed resources are relevant and improve patient understanding and knowledge

### Quality of studies

Eighteen out of the 24 papers were assessed as moderate to high quality. Three papers—two non-peer reviewed casebooks and a study protocol, were assessed as low quality. Another three papers were deemed low-moderate quality and consisted of another casebook, a study protocol and a qualitative case study. The latter was assessed as low quality due to unclear reporting. It is possible that the casebooks and study protocols scored poorly due to the lack of appropriate assessment tools for these types of publications. (see Table 3).

**Table 3:** Quality assessment

	Is there congruity between the stated philosophical perspective and the research methodology?	Is there congruity between the research methodology and the research question or objectives?	Is there congruity between the research methodology and the methods used to collect the data?	Is there congruity between the research methodology and the representation and analysis of the data?	Is there congruity between the research methodology and the interpretation of the results?	Is there a statement locating the researcher culturally or theoretically?	Is the influence of the researcher on the research, and vice-versa, addressed?	Are the participants, and their voices, adequately represented?	Is the research conducted according to current criteria or, for recent studies, and is there evidence of ethical approval by an appropriate body?	Do the conclusions drawn in the research report flow from the analysis, or interpretation, of the data?	Score (%)	High/Medium /Low quality
Cowdell 2020 [27]	Yes	Yes	Yes	Yes	Yes	Yes	Yes	Yes	Yes	Yes	100	High
Dal Mas 2020 [50]	Yes	Yes	unclear	Yes	Yes	No	No	Yes	No	Yes	60	Medium
Dent 2016 [36]	Unclear	Yes	Yes	Yes	Yes	No	No	Yes	Yes	Yes	70	High
Fonseka 2019 [43]	Yes	Yes	Unclear	Yes	Yes	No	No	Yes	No	Yes	60	Medium
Grindell 2020 [28]	Unclear	Yes	Yes	Yes	Unclear	No	Unclear	Yes	Yes	Yes	60	Medium
Heaton 2016 [29]	Unclear	Yes	Yes	Yes	Yes	No	No	Yes	Yes	Yes	70	High
IKT Casebook Volume 1 2019 [44]	Yes	Yes	Yes	Unclear	Unclear	No	No	Yes	Unclear	Yes	50	Medium
IKT Casebook Volume 3 2020 [45]	Unclear	Yes	Yes	NA	Unclear	No	No	Unclear	Unclear	Unclear	20	Low
Knowles 2018 [30]	Unclear	Yes	Yes	Yes	Yes	No	No	Yes	Yes	Yes	70	High
Law 2020 [31]	Yes	Yes	Yes	NA	NA	No	NA?	Yes	Yes	NA	50	Medium
Lewando-Hundt 2019 [32]	Yes	Yes	Yes	Yes	Yes	No	No	Yes	Yes	Yes	80	High
Livings 2020 [37]	Unclear	Yes	Yes	NA	NA	No	No	Yes	Yes	NA	40	Low
Miller 2016 [46]	Unclear	Yes	Yes	Unclear	Yes	No	Unclear	Yes	No	Yes	50	Medium
Milton [38]	Yes	Yes	Yes	Yes	Yes	No	No	Yes	Yes	Yes	80	High
Ospina- Pinillos 2018 [41]	Unclear	Yes	Yes	Yes	Yes	No	No	Yes	Yes	Yes	70	High
Ospina-Pinillos 2019 [39]	Unclear	Yes	Yes	Yes	Yes	No	No	Yes	Yes	Yes	70	High
Ospina-Pinillos 2020 [40]	Unclear	Yes	Yes	Yes	Yes	Unclear	Unclear	Yes	Yes	Yes	70	High
Reeve 2016 [33]	Unclear	Yes	Yes	Yes	Yes	No	No	Unclear	Yes	Yes	60	Medium
Revenas 2018 [48]	Yes	Yes	Yes	Yes	Yes	No	No	Yes	Yes	Yes	80	High
Thompson 2020 [47]	Unclear	Yes	Yes	NA	NA	No	No	Yes	Yes	NA	40	Low
TK2A Casebook 2019 [34]	Unclear	Yes	Yes	Yes	NA	Yes	Yes	No	Unclear	Unclear	40	Low
Wannheden 2020 [49]	Yes	Yes	Yes	Yes	Yes	No	No	Yes	Yes	Yes	80	High
Wolstenholme 2020 [35]	Unclear	Yes	Yes	Yes	Yes	No	No	Yes	Yes	Yes	70	High
Yanageneh	Unclear	Yes	Yes	Yes	Yes	No	No	Yes	Unclear	Yes	70	High

Low scoring are either research protocols and non peer reviewed casebooks for which there were no specific quality assessment tool available to use.

### Overview of Themes

**Fig. 2:**
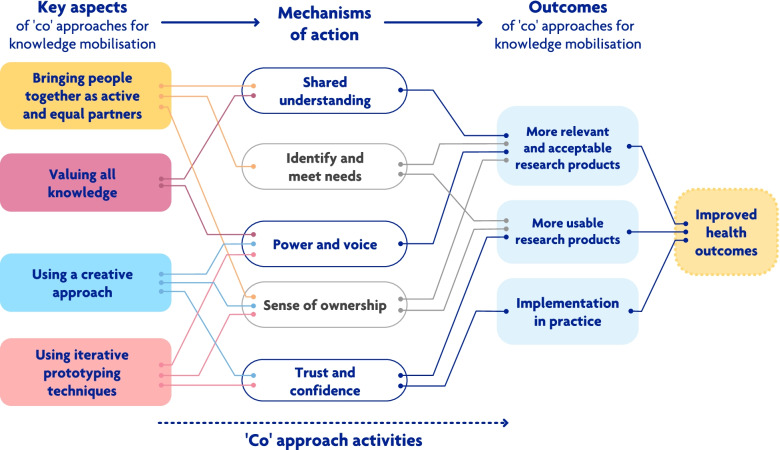
Overview of themes: key aspects, mechanisms of action, activities used and outcomes of ‘co’approaches for knowledge mobilisation in health conditions

Overall four themes were identified: 1. Key aspects of ‘co’approaches for knowledge mobilisation. 2. Mechanisms of action. 3. Activities used. 4. Outcomes of ‘co’approaches for knowledge mobilisation. The themes and their sub-themes, along with the relationships between them, are illustrated in Fig. 2.

### Key aspects of ‘co’ approaches for knowledge mobilisation

The aspects of ‘co’approaches that authors proposed as important to mobilise knowledge to improve the management of health conditions included: bringing people together as active and equal partners, valuing all knowledge, using a creative approach, and iterative prototyping techniques.

### Bringing diverse people together as active and equal partners

Forming collaborations between different stakeholders was considered critical [29, 32, 36, 38, 42, 47]. Authors believed that partnership working led to the sharing of goals [35], responsibilities and decision making throughout the process [27, 30, 31, 44, 47, 48]. Involving the right people in the ‘co’approach was considered to be central to knowledge mobilisation. For example, one study recognized that*:*

*‘involving all stakeholders can provide richer insights than involving patients or professionals alone’* [30].

Another proposed that by promoting inclusivity:

‘*meaningful egalitarian partnerships are formed between participants*’ [28].

Actively engaging stakeholders was identified as important [28, 31, 35, 36, 38], where they are:

‘*active agents not merely passive subjects or recipients of services’* [29].

### Valuing all knowledge

Authors acknowledged the existence of disparate types of knowledge in terms of research evidence, experience and opinions. They highlighted the need to include, recognise and understand all knowledge [27, 31, 32, 41, 44, 49] and place equal importance [29] on evidence-based research knowledge, clinical knowledge and experiential knowledge [27, 28, 36, 40, 47, 50]. Some authors suggested that ‘co’approaches offered an opportunity to generate, share and gain locally generated knowledge and experience from different sources [28, 30, 36, 48].

‘*Our approach is potentially efficient in making use of all available knowledge (scientific and ‘practical’); and potentially effective in being grounded in the reality and complexity of applied practice’* [33].

### Using a creative approach

Collaborative ways of working, inherent in ‘co’approaches, were deemed to be significantly different to the usual way of doing applied health research [29, 39]:

*‘the researchers and clinicians in some of the projects found that their experience of working in collaboration on the projects was different to how they had carried out research before (‘game changers’) and opened up new possibilities and capacity’* [29]*.*

Design and creative practice were recognised as a means to successfully bring the knowledge, skills, expectations and beliefs of heterogeneous groups of people together [28, 32, 34, 50]. Encouraging those involved to think and behave in different ways [29, 30] enhancing idea generation [39, 41].

Maintaining engagement of stakeholders was recognised as difficult. One study found that despite regular project meetings and media awareness campaigns they did not maintain engagement of key stakeholders through to implementation [36]. In contrast other studies [38, 41, 44] that favoured creative activities, felt that their design and participatory methods helped to engage diverse groups of people with varying goals, feelings and abilities. They perceived that their ‘co’approach helped retain engagement even within those groups who do not traditionally get involved in research [34, 35, 39, 50]:

‘*The research and development cycle that we employed in this study is an optimal methodology to engage, retain, and work more efficiently with hard-to-reach populations’* [39].

### Innovative, iterative and prototyping techniques

Many of the study authors proposed to use a flexible, iterative process to achieve successful knowledge mobilisation [27, 28, 30, 33, 35, 44, 46]. For example, the iterative PaCER process in one study allowed learning from participants in each phase to inform the next [46]. Another felt that flexibility was essential to adapt knowledge to context in a complex dynamic system such as healthcare [33].

Iterative prototyping, often used in design practice, was adopted in a number of studies [28, 30, 31, 34, 35, 39–41]. Prototyping was considered useful for turning knowledge into practical, tangible objects [28, 34, 35]. For example, one study used quick, easy and cheap, low fidelity prototypes to generate iterative cycles of feedback and development [28]. In other studies, visual design artefacts such as videos, drawings and sketches were used [28, 31, 34, 39–41, 50]. Authors felt that ideas could be quickly communicated in this way in simple, understandable forms making knowledge more accessible [28, 30, 34, 50].

Expert facilitation of these varied activities was considered to be crucial to their success. The use of independent facilitators was found to be successful [34, 35, 43]. They appeared to reduce anxieties regarding participation and encourage open and honest contributions [34, 43]:

*‘Having a design facilitator enabled visualisation of thoughts and ideas as they arose. This allowed real time synthesis of occurring knowledge, for example through drawings, which was presented in a form that was easy to understand and which accurately represented participant’s views’* [28]*.*

Alternatively training could be given to enable researchers to facilitate these activities successfully [30].

### Mechanisms of action

‘Co’approaches were considered to achieve knowledge mobilisation through a number of mechanisms of action directly related to the key aspects described. Study authors considered that bringing people together as active partners, valuing all forms of knowledge, using a creative approach and iterative prototyping techniques, could facilitate a shared understanding of the problem and identify important needs and how to meet them, thereby balancing power differentials, offering a sense of ownership, and engendering trust and confidence in solutions.

### Shared understanding

Authors reported engaging multiple stakeholders in the process could identify wider perspectives and contexts and contribute to a shared understanding of the problems and potential solutions [27, 28, 30, 32, 33, 38, 40, 43, 46].

Using design artefacts to communicate participants’ thoughts and feelings could facilitate the generation of knowledge and develop a mutual understanding of what was important to stakeholders [28, 30, 34, 50]. The use of personas [28, 30, 34, 35] and scenarios [30, 34] were thought to help distance participants from their own positions and prevent a ‘them and us’ dynamic developing [30].

*‘The persona seemed to be particularly powerful for the professional group and prompted a focus on considering the “whole person” experience that the attendees said they may not have considered otherwise’* [30]*.*

This meant that outputs were a consensus between participants, considering all perspectives, rather than the product of situated assumptions, such as what health care professionals think patients want or need [30].

### Identify and meet needs

Authors described that by bringing diverse groups of people together, pooling their ‘creative assets’ [29], and considering and valuing their different types of knowledge, expertise and perspectives, they could produce outputs that were tailored to everyone’s needs [29, 32, 38, 41, 42, 46, 47, 50]. They felt that by including people with lived experience of a health condition in the process they were able to contribute their unique perspectives and ideas [29, 32, 35, 48] and the research addressed the areas that patients felt were most important [36, 43]. This challenged the traditional medical model which assumes the clinician knows best [27, 43].

*‘because clinical guidelines are often developed using the medical model where clinicians are considered to possess knowledge and expertise over what is best for the patient’* [43]*.*

By valuing diverse evidence and knowledge, authors perceived that complex systems and services, such as those in healthcare, could be better understood as no one individual could understand them completely [33, 35]. In this way ‘co’approach outputs could attend and align to context [28, 29, 34, 38] including wider organisational factors [29]. Authors felt that using creative and iterative prototyping techniques allowed them to challenge and refine ideas into practical concepts that were fit for purpose and more likely to meet stakeholder needs [30, 38].

### Balancing power and voice

Authors felt that balancing power and voice of those involved aided knowledge mobilisation. Authors felt this was achieved in various ways. Two studies suggested that giving clinicians, patients and the public a more active role in the whole research process meant that they felt valued and had a more equal role [29, 45]. In other studies, involving people with lived experience meant their voices were listened to and valued [45, 46]. One study used research based theatre to achieve this [32]:

*‘Theatre makers on the panel were able to explain the process of developing research based Theatre and by doing so revealed how the voices of research participants were respected and heard’* [32]*.*

A number of studies found that their ‘co’approaches challenged traditional relationships between patients and doctors [28, 34, 35, 41, 50] or blurred practice and academic boundaries [28, 33–35]:

*‘The discussion was not led by power players such as scientists or surgeons that could have used their status to lead the discussion’* [50]*.*

Several studies [28, 34, 35, 39, 41, 50] found that the use of creative activities had a positive influence on group dynamics. For example one study felt that their design-led activities enabled participants to:

‘*share and express themselves in an inclusive environment using a common language.*’ [28].

Another author felt that power hierarchies could be flattened and more voices heard by making ideas tangible [34]. Creative activities were found to be helpful in engaging people ‘*who might otherwise have struggled to participate’* [34] and contribute to the process, such as people with verbal communication problems or lower literacy levels [34, 35]. Skilled facilitation was recognised as important in order to manage the power asymmetries found in heterogenous groups of people [48].

### Sense of ownership

Authors anticipated that knowledge could be shared and generated by bringing people together to form collaborative partnerships, creating a sense of ownership and common purpose [28, 44] that would help reduce the research to practice gap [36]. Ownership was reinforced by considering context, implementation and by valuing all stakeholder knowledge [28, 29, 34]:

*‘These include developing strong cross-sector partnerships with stakeholders to co- create and share emerging knowledge, integrating and utilizing all stakeholders’ relevant expertise and experience and promoting a sense of ownership and common purpose’* [44]*.*

### Trust and confidence

Authors identified that stakeholders would have more trust and confidence in the final outputs because their needs were identified, a shared understanding was gained, power and voice was attended to and a sense of ownership was achieved [28, 46]. A number of authors deemed their outputs to be more credible, relevant, practical, realistic, and trustworthy, because of their ‘co’approach [28, 29, 33, 34, 39, 40, 42, 43, 46, 48].

*‘This experience only confirmed their view that it was important to include representatives of all the relevant professionals in the process of building a model, to make it sufficiently realistic and trustworthy, and to increase the chances of the results being accepted by them and acted upon’* [29]*.*

### Activities used in ‘co’approaches

Authors used a range of activities, regardless of the term used for their ‘co’approach, in order to achieve the mechanisms of action discussed. It is useful to document these because often researchers rely on research methods when other activities can help to achieve these mechanisms (see Table 4). For example a number of studies included creative activities drawn from design, such as drawing and sketching, personas, journey maps and prototyping [27, 28, 30, 31, 34, 35, 38–41, 48–50]. Some used the amalgamation of interview and focus group data to inform their ‘co’approach process [42, 44, 46]. Others were co-production or co-design of a whole research project [29, 44–46]. Prioritisation and consensus techniques were common, including nominal group and Delphi techniques [27, 31, 34, 37, 43, 47–49]. One study used a writing committee [43] and others used meetings and discussion groups [27, 32, 36, 37, 44, 49]. Generally some form of workshop was common.

**Table 4:** Type of activity used within ‘co’approaches

Method	Activity used by authors in this review	Definition/description from general literature or from the papers in the review
**Research methods**	**(Semi-structured) interviews **[38, 42, 46, 50]	‘Where the researcher has a list of questions or specific topics to be asked using an interview guide. Questions do not have to be followed as per the guide and new questions can be asked as the researcher picks up on things the interviewee says.’ [51]
	**Focus groups **[44, 46, 48, 49]	‘A form of group interview with a number of participants and a moderator. Questions follow a fairly tightly defined topic with a focus on interaction between the group.’ [51]
	**Observations **[44]	‘Immersion in a group for a period of time observing behaviour, listening to what is said and asking questions.’ [51]
	**Surveys/feedback forms **[32, 42, 44]	‘Respondents read and answer a series of questions themselves.’[51]
	**Qualitative enquiry **[47]	‘Qualitative inquiry refers to “a broad approach” that qualitative researchers adopt as a means to examine social circumstances. The inquiry is based on an assumption which posits that people utilize “what they see, hear, and feel” to make sense of social experiences. The meanings and interpretations of the participants are the essence of qualitative inquiry.’ [52]
	**Guideline/literature appraisal **[42]	*‘a synthetic review and summary of what is known and unknown regarding the topic of a scholarly body of work, including the current work's place within the existing knowledge.’ *[53]
**Prioritisation and consensus methods**	**Prioritisation/ranking **[27, 31, 34, 43]	‘At the point of defining which of several ideas we should take forward. The visual act of assessing for impact and feasibility can be done in a participatory and visual way.’ [34]
	**Consensus **[37, 42]	‘Consensus methods provide a means of harnessing the insights of appropriate experts to enable decisions to be made.’ [54]. They are ‘a way to gather general agreement on topics that do not yet have empirical evidence to support future decisions or actions; often, these topics are ambiguous or controversial. Consensus methods can also be used as a way to forecast future events or create decision protocols.’ [55]
	**Nominal Group technique **[48, 49]	‘The purpose is to generate ideas, which are discussed and ranked by the group. The group is 'nominal' to the extent that it is highly controlled and discussion is allowed only during the later stages of the group process. It was originally designed to avoid the problems associated with traditional interacting groups.’ [56]
	**Delphi technique **[47]	‘a group of 'expert' participants are sent a postal questionnaire about the area of interest. Responses are then sent to a panel who collate and assess the participants views, which are then fed-back to the participants, usually in the form of a more structured questionnaire. The participants return their second responses to the panel and the process is repeated for as many rounds as necessary to achieve either a consensus on the subject under study, or allow a full understanding of opposing perspectives to be achieved.’ [56]
**Research co-production/co-design**	**Engaging all stakeholders throughout research project** [29, 44–46]Joint leading project team, refine scope, develop research questions, develop and review content, protocol development and adaptation, collect data and reflect on findings- patients as researchers throughout project, assist in implementation	‘co-producing a research project is an approach in which researchers, practitioners and the public work together, sharing power and responsibility from the start to the end of the project, including the generation of knowledge.’ [12] Integrated Knowledge translation is a specific form of research co-production. It is described as ‘a model of collaborative research, where researchers work with knowledge users who identify a problem and have the authority to implement the research recommendations.’ [57]
**Creative methods **	**Making activities **[31, 34]	‘used as vehicles for collectively (e.g. designers and co-designers together) exploring, expressing and testing hypotheses about future ways of living.’ [58]
	**Warm up activities **[28, 34, 35, 43]	‘Not just ice breakers warm up activities focus on supporting individuals to recognise their own unique ability to contribute to creative process regardless of background or role in project’. [34]
	**LEGO® SERIOUS PLAY® **[31]	‘Based on research which shows that hands-on, minds-on learning produces a deeper, more meaningful understanding of the world and its possibilities, the LEGO® SERIOUS PLAY® methodology deepens the reflection process and supports an effective dialogue – for everyone in the organization.’ [59] The techniques ‘stimulate ideas and creativity, work with metaphor, symbolism and association and are highly democratic and non-hierarchical.’ [60]
	**Sketching and drawing **[31, 39–41, 46, 50]	‘Sketching is a rapidly executed freehand drawing that is not usually intended as a finished work. It may serve a number of purposes: it might record something that the artist sees, it might record or develop an idea for later use or it might be used as a quick way of graphically demonstrating an image, idea or principle’ [61]. Drawing as a participatory research method ‘relies on researcher-participant collaboration to make meaning of the drawing.’ [62]
	**Personas **[30, 34, 35, 38]	Fictional characters representing a particular group and their interests and needs. [63, 64]. They can be used ‘to visually represent peoples experiences through characters that allow critical distance from participants’ own experience.’ [34]
	**Maps/user journeys **[28, 34, 35, 38]	‘A vivid and structural visualisation of a service users experience. Touchpoints, where users interact with the service, are often used to construct a ‘journey’/engaging story based on their experience.’ [64] ‘It may show pitfalls and opportunities and support choices of route and targets.’ [63] They can be ‘useful when the journey (service or user) is usually not visible to all actors. The visual aspect allows all participants to contribute adding new lines or items.’ [34]
	**Posters **[34, 35]	Can be used to ‘summarise progress to date or remind participants of the goal of the workshop/project.’ [34]
	**Storyboards **[34]	‘A series of drawings or pictures that visualise a particular sequence of events. May include a common situation where a service is used or the hypothetical implementation of a new service prototype’ [64]. They often’ resemble a comic strip with captions.’ [63]They can be used to ‘visually represent either problems or solutions that allow participants to suggest different key steps or endings that might lead to a better outcome.’[34]
	**Scenarios **[30, 48]	‘A story, typically of how people perform a part of their lives or an interaction with a product or service.’ [63]
	**Role play **[37]	‘The physical acting out of scenarios and prototypes in a situation that resembles a theatre rehearsal.’ [64]
	**Research based theatre** (post performance panel discussions) [32]	‘Research-based Theatre provides a multi-disciplinary platform that enables the impact of original research to extend its reach beyond academic publications and presentations.’ [32]‘Experiencing live Theatre performance created from research findings deepens understanding and allows for learning through cognitive and emotional engagement and debate of complex and contested issues during post-show discussion.’ [65]
	**Ideation **[30, 35, 39–41, 48]	‘The process of generating ideas.’ [63] ‘Ideation techniques are used to structure and inspire group brainstorming sessions. Usually simple exercises which can be used to stimulate group discussion whilst providing structure within which to work.’ [64]
	**Blue sky thinking **[30, 35, 39–41, 48]	Creative ideas that are not limited by current thinking or beliefs. [66]
	**Prototyping **[28, 30, 31, 34, 35, 38–41, 44, 45, 48, 49]	‘Artifacts created to explore a (design) question or to express a conceptual design, used to evaluate ideas with users’ [63]. They are ‘physical manifestations of ideas or concepts. They range from rough (giving the overall idea only) to finished (resembling the actual end result). To give form to an idea, and to explore technical and social feasibility. Co-designers create the prototypes to envision their ideas and to display and to get feedback on these ideas from other stakeholders.’ [58]They make ‘a process or idea tangible and can be 2D (sketch or video) or 3D (proof of concept visualisation or fully working). They are good for communicating ideas and gathering feedback.’ [34]
	**Trigger films **[30]	A method used in Experience Based Co-Design that involves making ‘a video film of ‘touchpoints’ (where interaction with a service occurs) from patient experience interviews that exemplify good or bad experiences of a service.’ [67]
	**Future Workshops** [30] (Personas , scenarios- described in creative methods)	Future workshop is a method that aims to have stakeholders design their desired future, avoiding constraints imposed by experts or organizations. [68]
	**‘Talking points’ **[42]	‘Talking points are part of the HealthTalk/DIPEx patient experience approach which are well-established methods of qualitative research which are based on the pioneering work the Health Experiences Research Group in the Nuffield Department of Primary Care Health Sciences at University of Oxford.’ [69]Talking points are described as a presentation of themes through video, audio or text format. [42]
**Other**	**A writing committee **[43]	Training to support writing and resources to help writing and amending a guideline.[43]
	**Improvement – in practice- in context **[33]	‘through the generation of practice-based evidence, with researchers and clinicians working together to co-construct and evaluate a new account of practice.’ [33]
	**Note cards/post cards **[27, 35, 48, 49]	
	**Meetings **[36, 44]	
	**Teleconference**s [44]	
	**Presentations** [37, 42]	

### Achieving outcomes

Few of the included studies measured outcomes. Authors tended to describe the outcomes they believed they were more likely to achieve. These included more relevant research products, more usable knowledge, outputs more likely to be implemented in practice, and improved health.

### More accessible, relevant and acceptable knowledge mobilisation products

Two authors perceived that their ‘co’approach helped overcome the problem of research and research findings seeming inaccessible and irrelevant to non-academic audiences [28, 35]. Other authors felt their use of visualisations and design artefacts improved the accessibility of knowledge by simplifying complex concepts [28, 30, 35, 39, 50]. Making research and its findings more accessible and relevant was considered an important outcome [35, 43, 47].

*‘The participation of end users in the design process ensured that the prototype was accessible to individuals of varying literacy levels with a range of cultural differences’* [39]*.*

Authors indicated that by using collaborative approaches they could produce more engaging, functional, practical and acceptable products [28, 37, 39–42]. Findings from user testing of prototype functionalities for an e-mental health management system supported this view [39–41]. Authors felt that their participatory ‘co’approach could: ‘*help ensure the end product meets everyone’s needs; improve usability; and increase engagement of users’* [41] and ‘*could result in better products that are more functional in real-life settings’* [40]*.*

### More usable knowledge products

A number of authors felt their ‘co’approach produced outputs with potential to be useful and useable in practice [28–30, 33, 34, 39, 42, 43]. Several felt that their outputs were more likely to be accepted and therefore more likely to be acted upon and used, leading to successful changes in practice [28, 29, 33, 34, 44, 45, 47, 48]. Authors felt that outputs would be fit for purpose in the real world because their ‘co’approach ensured cultural and contextual factors were captured and used to inform their generation [28, 33, 34, 40, 43, 48].

*‘Including people with lived experience in guideline development can aid improved understanding of treatment options, greater involvement in health care decision making, and increased satisfaction in primary and secondary health care. This model can be used to to ultimately produce a product that has real‐world utility for patients and their families’* [43]*.*

Few studies carried out formal evaluation of their outputs, however data collected in four studies indicated that the process could produce useful and easy to use outputs [35, 42, 44, 50].

### Implementation in practice

Authors proposed that because their research was more relevant, acceptable and usable it was more likely to be implemented in practice. A number of studies provided insights into how their outputs had been implemented and impacted on clinical practice both locally and nationally [29, 33, 35, 36, 44, 46].

*“because of our adoption of the Toolbox, our implementing clinicians have assessed chronic pain in over 70% of their pediatric patients who may not have otherwise discussed their chronic pain*’’ [44].

Two casebooks used the IKT approach to ensure research outputs were more implementable [44, 45]. Other studies found that prototypes incorporating culturally and contextually specific information had the potential to aid implementation [28, 34, 39–41]. most of the studies in this review produced outputs that required further refinement before being ready to be implemented [48].

It was acknowledged that implementation and sustained engagement with outputs was challenging. In order to achieve sustainability and long term impact after research teams departed local champions were required to continue to drive implementation forward [36].

### Improved health

None of the included studies in this systematic review undertook an in depth post implementation evaluation nor did they measure or report on specific health outcomes. Many of the authors aspired to, and in some cases reported, the goal of improving healthcare outcomes and quality of care [28, 30, 34, 37, 43, 46, 50]. However, these claims were not based on robust evaluation data and evaluation methods were not clearly reported. A number of authors felt improving the relevance [40, 41, 43, 46], acceptability [37, 40] and usability [40, 41] of outputs would improve outcomes or quality of care.

*‘the development of a codesigned conservative model of care involving patients, clinical staff, members of the public and other stakeholders is more likely to be accepted by both providers and users, resulting in a higher rate of stakeholder satisfaction, continuous improvement and a reduced failure risk’* [37]*.*

Other studies demonstrated actual changes in practice as a result of introducing the co-designed outputs. These included improved consistency in clinician assessment and identification of patient problems that were previously missed [44], changes to clinical pathways [29], fewer hospital visits and admissions [44] and a reduction in the number of patients who failed to attend appointments [35]. Additional positive outcomes such as, patient satisfaction were either shown or perceived to be possible [33, 43].

## Discussion

From the 24 included studies authors’ main reasons for choosing a ‘co’approach were: 1. Bringing people together. 2. valuing all knowledge. 3. To produce more relevant research products. 4. To improve health outcomes. These were achieved through several mechanisms, such as identifying and meeting all stakeholders’ needs and enabling trust and confidence in the outputs. However, there was little evidence that these approaches improved health because of the lack of robust evaluation of the interventions produced. Despite this, the findings provide useful insights into how ‘co’approaches might mobilise knowledge in health condition management and they are aligned with the five principles for co-production described by a leading research funder in the UK [12]. The NIHR [12] propose the principles of: 1. Sharing power. 2. Including all perspectives and skills. 3. Respecting and valuing all knowledge. 4. Reciprocity and 5. Building and maintaining relationships. Our review builds on these principles by highlighting activities researchers use to achieve them, further key aspects and mechanisms of action, and the relationships between them. For example, sharing of power may be facilitated if the ‘co’approach brings people together as active partners and uses creative activities. Building and maintaining relationships may be promoted by using iterative prototyping techniques. The findings from this review suggest that the process of developing adaptable, visible and tangible outputs helps participants see that their knowledge and ideas have been heard and valued. Participants may have more trust in the process and reciprocity achieved by producing relevant and acceptable outputs that meet everyone’s needs.

Langley et al.’s 2018 ‘collective making’ knowledge mobilisation model [70] specifically considers the influences of creative practices. The authors propose that their ‘collective making’ ‘co’approach influences the participants involved, the knowledge being mobilised and implementation in a number of ways [70] similar to the findings in this review. For example, influencing participants through balancing power and voice and enabling articulation of complex concepts; influencing knowledge through accessing, sharing and valuing different types of knowledge; influencing implementation through creating a sense of ownership and trust in the co-created outputs. Our review complements this model and highlights that some researchers believe similar benefits can be gained without the use of creative activities. This review demonstrates that there is no ‘one size fits all’ approach. All three ‘co’approaches, that is co-production, co-design and co-creation, were used in the studies in this review utilising a variety of activities, from research methods such as interviews and focus groups to workshops using creative activities drawn from design.

### Strengths and limitations

This is the first systematic review of ‘co’approaches for knowledge mobilisation for the management of health conditions and included a large number of studies. There were however some limitations. First, there was a lack of studies that had formally evaluated the outputs of their ‘co’approach. A review focused explicitly on the effectiveness of interventions for knowledge mobilisation might have identified more relevant literature than our review. Second, the inclusion/exclusion criteria may have excluded some studies. For example, some collaborative and participatory research that could be deemed to sit under the co-production umbrella, such as studies using an IKT approach, were not included because they did not explicitly describe their approach as co-production, co-design or co-creation. The focus of this systematic review was on these three commonly used terms specifically and knowledge mobilsation. Therefore on reflection, we think that this exclusion criterion was necessary in order to make some sense of this diverse and complex field. Third, the elasticity of the term knowledge mobilisation in the healthcare literature meant the inclusion criteria for this term was broader and encompassed other terms such as knowledge exchange and evidence into practice. This meant that there was room for interpretation by the reviewers which may have led to reviewer bias. Fourth, the lack of use of MeSH terms may have reduced the number of search results meaning some potentially relevant papers may have been missed. Finally, the lead reviewer conducted the majority of the screening process and was the author or co-author of some of the included papers. The bias of the first author was minimised to some degree by working closely with a second reviewer and discussions with other authors of the review.

### Conclusions and Implications for future research

This systematic review suggests that ‘co’approaches show promise in achieving successful knowledge mobilisation to improve the way health conditions are managed. However, the findings relied heavily on authors’ beliefs, with only some supporting evidence for short term outcomes such as producing acceptable outputs. There is a need for robust evaluation to ascertain the extent to which ‘co’approaches can produce improved health outcomes. A systematic review that evaluates outputs from ‘co’approaches versus those produced using alternative approaches in a diverse range of settings is recommended to assess whether the former are more likely to achieve knowledge mobilisation and improved outcomes.

Finally, undertaking research using ‘co’approaches is no easy task and it is a common criticism within the literature that authors rarely report their activities in detail nor the steps they have taken to adapt their methods to align with the key principles of ‘co’approaches [13]. The themes diagram in this review is a form of logic model [71] displaying the pathways through which ‘co’approaches might achieve desired outcomes. This could be used as a framework to help people using ‘co’approaches align their chosen activities to the key aspects and mechanisms, as identified within this review, and the principles of ‘co’approaches articulated elsewhere [12, 70]. This will aid transparency in reporting and potentially improve an intervention’s chance of achieving successful knowledge mobilisation.

## Supplementary Information

**Additional File 1.** 

## Data Availability

The datasets used and/or analysed during the current study are available from the corresponding author on reasonable request.
