# Supplementary material for: The use of co-production, co-design and co-creation to mobilise knowledge in the management of health conditions: a systematic review
Source: BMC Health Serv Res. 2022 Jul 7;22:877. doi: 10.1186/s12913-022-08079-y (PMC9264579; doi:10.1186/s12913-022-08079-y)
Supplement: Supplementary file 1 — Additional File 1. [file 12913_2022_8079_MOESM1_ESM.docx]

Database: Embase <1988 to April 30 2021

Search Strategy:

--------------------------------------------------------------------------------

1 co-production.mp. [mp=title, abstract, heading word, drug trade name, original title, device manufacturer, drug manufacturer, device trade name, keyword heading word, floating subheading word, candidate term word]

2 coproduction.mp. [mp=title, abstract, heading word, drug trade name, original title, device manufacturer, drug manufacturer, device trade name, keyword heading word, floating subheading word, candidate term word]

3 co production.mp. [mp=title, abstract, heading word, drug trade name, original title, device manufacturer, drug manufacturer, device trade name, keyword heading word, floating subheading word, candidate term word]

4 co-produc*.mp. [mp=title, abstract, heading word, drug trade name, original title, device manufacturer, drug manufacturer, device trade name, keyword heading word, floating subheading word, candidate term word]

5 coproduc*.mp. [mp=title, abstract, heading word, drug trade name, original title, device manufacturer, drug manufacturer, device trade name, keyword heading word, floating subheading word, candidate term word]

6 co produc*.mp. [mp=title, abstract, heading word, drug trade name, original title, device manufacturer, drug manufacturer, device trade name, keyword heading word, floating subheading word, candidate term word]

7 co-design.mp. [mp=title, abstract, heading word, drug trade name, original title, device manufacturer, drug manufacturer, device trade name, keyword heading word, floating subheading word, candidate term word]

8 codesign.mp. [mp=title, abstract, heading word, drug trade name, original title, device manufacturer, drug manufacturer, device trade name, keyword heading word, floating subheading word, candidate term word]

9 co design.mp. [mp=title, abstract, heading word, drug trade name, original title, device manufacturer, drug manufacturer, device trade name, keyword heading word, floating subheading word, candidate term word]

10 co-creat*.mp. [mp=title, abstract, heading word, drug trade name, original title, device manufacturer, drug manufacturer, device trade name, keyword heading word, floating subheading word, candidate term word]

11 cocreat*.mp. [mp=title, abstract, heading word, drug trade name, original title, device manufacturer, drug manufacturer, device trade name, keyword heading word, floating subheading word, candidate term word]

12 co creat*.mp. [mp=title, abstract, heading word, drug trade name, original title, device manufacturer, drug manufacturer, device trade name, keyword heading word, floating subheading word, candidate term word]

13 1 or 2 or 3 or 4 or 5 or 6 or 7 or 8 or 9 or 10 or 11 or 12 (233)

14 knowledge mobili*.mp. [mp=title, abstract, heading word, drug trade name, original title, device manufacturer, drug manufacturer, device trade name, keyword heading word, floating subheading word, candidate term word]

15 knowledge transl*.mp. [mp=title, abstract, heading word, drug trade name, original title, device manufacturer, drug manufacturer, device trade name, keyword heading word, floating subheading word, candidate term word]

16 knowledge utili*.mp. [mp=title, abstract, heading word, drug trade name, original title, device manufacturer, drug manufacturer, device trade name, keyword heading word, floating subheading word, candidate term word]

17 knowledge exchange.mp. [mp=title, abstract, heading word, drug trade name, original title, device manufacturer, drug manufacturer, device trade name, keyword heading word, floating subheading word, candidate term word]

18 knowledge uptake.mp. [mp=title, abstract, heading word, drug trade name, original title, device manufacturer, drug manufacturer, device trade name, keyword heading word, floating subheading word, candidate term word]

19 knowledge to action.mp. [mp=title, abstract, heading word, drug trade name, original title, device manufacturer, drug manufacturer, device trade name, keyword heading word, floating subheading word, candidate term word]

20 knowledge to practice.mp. [mp=title, abstract, heading word, drug trade name, original title, device manufacturer, drug manufacturer, device trade name, keyword heading word, floating subheading word, candidate term word]

21 evidence based practice.mp. [mp=title, abstract, heading word, drug trade name, original title, device manufacturer, drug manufacturer, device trade name, keyword heading word, floating subheading word, candidate term word]

22 14 or 15 or 16 or 17 or 18 or 19 or 20 or 21

23 13 and 22

Database: Ovid MEDLINE(R) ALL <1946 to April 30, 2021>

Search Strategy:

--------------------------------------------------------------------------------

1 co-production.mp.

2 coproduction.mp.

3 co production.mp.

4 co-design.mp.

5 codesign.mp.

6 co design.mp.

7 co-create.mp.

8 cocreate.mp.

9 co create.mp.

10 co-creation.mp.

11 cocreation.mp.

12 co creation.mp.

13 or/1-12

14 knowledge mobil*.mp.

15 knowledge transl*.mp.

16 knowledge translation.mp.

17 knowledge utili*.mp.

18 knowledge utilisation.mp.

19 knowledge transf*.mp.

20 knowledge transfer.mp.

21 knowledge uptake.mp.

22 evidence based practice.mp.

23 knowledge to action.mp.

24 knowledge to practice.mp.

25 or/14-24

26 13 and 25

| Web of Science all databases) 1970-April 30 2021 |  |  |  | Display |
| --- | --- | --- | --- | --- |
| **TOPIC: ("co -production")**  **TOPIC: ("co production")**  **TOPIC: ("coproduction")**  **TOPIC: ("co-produc*")**  **TOPIC: ("coproduc*")**  **TOPIC: ("co produc*")**  **TOPIC: ("co-design")**  **TOPIC: ("codesign")**  **TOPIC: ("co design")**  **TOPIC: ("co-creat*")**  **TOPIC: ("cocreat*")**  **TOPIC: ("co creat*")**  **#12 OR #11 OR #10 OR #9 OR #8 OR #7 OR #6 OR #5 OR #4 OR #3 OR #2 OR #1**  **TOPIC: ("knowledge mobil*")**  **#14 AND #13**  **TOPIC: ("knowledge transl*")**  **#16 AND #13**  **TOPIC: ("knowledge utili*")**  **#18 AND #13**  **TOPIC: ("knowledge exchange")**  **#20 AND #13**  **TOPIC: ("knowledge uptake")**  **#22 AND #13**  **TOPIC: ("knowledge to action")**  **#24 AND #13**  **TOPIC: ("knowledge to practice")**  **#26 AND #13**  **TOPIC: ("evidence based practice")**  **#28 AND #13**  Database: CINAHL(EBSCOHost)  1981- April 30 2021   \| S1 \| TX co-produc* \|  \|  \| Display \| \| --- \| --- \| --- \| --- \| --- \|  \| S2 \| TX coproduc* \|  \|  \| \| --- \| --- \| --- \| --- \| \| S3 \| TX co produc* \|  \|  \| \| S4 \| TX codesign* \|  \|  \| \| S5 \| TX co-design* \|  \|  \| \| S6 \| TX co design* \|  \|  \| \| S7 \| TX co-creat* \|  \|  \| \| S8 \| TX co creat* \|  \|  \|   S9 TX cocreat*  S10 S1 OR S2 OR S3 OR S4 OR S5 OR S6 OR S7 OR S8 OR S9   \| S11 \| knowledge mobili* \|  \|  \| \| --- \| --- \| --- \| --- \| \| S12 \| knowledge transl* \|  \|  \| \|  \|  \|  \|  \| \| S13 \| knowledge utili* \|  \|  \| \| S14 \| knowledge exchange \|  \|  \| \| S15 \| knowledge uptake \|  \|  \| \| S16 \| evidence based practice \|  \|  \| \| S17 \| knowledge to action \|  \|  \| \| S18 \| knowledge to practice \|  \|  \| \| S19 \| S11 OR S12 OR S13 OR S14 OR S15 OR S16 OR S17 OR S18)) AND (S9 AND S19)) AND (S10 OR S11 OR S12 OR S13 OR S14 OR S15 OR S16 OR S17 OR S18 \|  \|  \|  \| S20 \| S10 AND S19 \|  \|  \| 99 \| \| --- \| --- \| --- \| --- \| --- \| \|  \|  \|  \|  \| Display \| |  |  |  | Display |
